# Two-Photon Excitation STED Microscopy with Time-Gated Detection

**DOI:** 10.1038/srep19419

**Published:** 2016-01-13

**Authors:** Iván Coto Hernández, Marco Castello, Luca Lanzanò, Marta d’Amora, Paolo Bianchini, Alberto Diaspro, Giuseppe Vicidomini

**Affiliations:** 1 Nanoscopy, Nanophysics, Istituto Italiano di Tecnologia, Via Morego 30, 16163 Genoa, Italy; 2 Department of Physics, University of Genoa, Via Dodecaneso 33, 16146, Genoa, Italy; 3 Department of Informatics, Bioengineering, Robotics and Systems Engineering, University of Genoa, Via Opera Pia 13, 16145, Genoa, Italy; 4 Nikon Imaging Center, Istituto Italiano di Tecnologia, Via Morego 30, 16163 Genoa, Italy

## Abstract

We report on a novel two-photon excitation stimulated emission depletion (2PE-STED) microscope based on time-gated detection. The time-gated detection allows for the effective silencing of the fluorophores using moderate stimulated emission beam intensity. This opens the possibility of implementing an efficient 2PE-STED microscope with a stimulated emission beam running in a continuous-wave. The continuous-wave stimulated emission beam tempers the laser architecture’s complexity and cost, but the time-gated detection degrades the signal-to-noise ratio (SNR) and signal-to-background ratio (SBR) of the image. We recover the SNR and the SBR through a multi-image deconvolution algorithm. Indeed, the algorithm simultaneously reassigns early-photons (normally discarded by the time-gated detection) to their original positions and removes the background induced by the stimulated emission beam. We exemplify the benefits of this implementation by imaging sub-cellular structures. Finally, we discuss of the extension of this algorithm to future all-pulsed 2PE-STED implementationd based on time-gated detection and a nanosecond laser source.

Since its first demonstration^1^, stimulated emission depletion (STED) microscopy^2^ has migrated from a simple bi-dimensional (xy) super-resolved microscopy technique to a live-cell and multi-dimensional technique^3^,^4^. This stems from the compatibility of the key phenomenon employed in this method, namely switching fluorophores off transiently by stimulated emission (SE), with most of the microscopy developments of the last decades. Indeed, STED microscopy has been successfully extended to the axial (z)^5^,^6^, spectral (λ)^7^,^8^ and temporal (t)^9^ dimension; it has been combined with lifetime imaging (τ)^10^, fluorescence-correlation-spectroscopy (FCS)^11^,^12^, fluorescent proteins staining^13^ and other recent probes for live-cell imaging^14^,^15^.

Two-photon excitation (2PE) microscopy^16^,^17^ is a noteworthy alliance with STED microscopy. *De facto*, the combination of the strengths of these two methods (2PE-STED) opened new perspectives for super-resolution imaging of thick specimens^18^. The first reliable implementations of 2PE-STED microscopy used a mode-locked Ti:Sapphire laser for the 2PE (excitation beam) and a continuous-wave (CW) laser for the SE (STED beam)^19^,^20^,^21^,^22^. The use of a CW laser as the STED beam avoids laser synchronization, offers a wide range of wavelengths and mitigates the cost, but the lower peak intensity provided by a CW laser, with respect to a pulsed laser, limits the spatial resolution performance^23^,^24^. The implementation of 2PE-STED microscopy with a pulsed STED beam (2PE-pSTED) overcame this limitation but complexity and cost both significantly increased^25^,^26^,^27^. At present, the 2PE-pSTED implementation needs the synchronization of two mode-locked Ti:Sapphire lasers in order to provide enough peak intensity for the 2PE and STED beams. Because of the need for an optical parameter oscillator^25^,^27^, the technical sophistication and the cost further increase when working with fluorophores in the visible range, such as the green or yellow fluorescent proteins (GFP and YFP). One solution that works with a pulsed STED beam and lowers cost and complexity is the single-wavelength- (SW-) 2PE-STED implementation^28^,^29^,^30^. In stark contrast to the 2PE process, the SE process is a single-photon process; hence by controlling the width of the pulses it is possible to obtain 2PE (hundreds femtosecond pulse-width) and SE (hundreds picosecond pulse-width) with the very same wavelength, same beam and same laser. However, the use of the same wavelength prevents the simultaneous optimization of the SE and the two-photon absorption cross-sections, which limits the generality of this implementation.

Here we report on a novel 2PE-STED microscopy architecture based on time-gated detection. It has been shown that the ability to switch-off fluorophores with a STED beam operating in CW, effectively improves if the fluorescence signal is collected with a delay (namely *T*_g_) from the fluorophore excitation events^24^,^31^,^32^. It is important to note that the use of time-gated detection does not improve the fluorescent quenching *per se*, namely the fluorophore’s probability of spontaneous de-excitation does not change, but the fraction to which the detected fluorescence signal is suppressed by the STED beam increases. This property decided the basis for a popular (one-photon excitation) STED system, usually referred to as a gated CW-STED (gCW-STED) microscope^32^,^33^,^34^,^35^. Because this implementation requires a pulsed excitation laser and a time-gated detection, the complexity and cost, compared to a classical CW-STED implementation, increases. However, no laser synchronization is introduced and the large portfolio of wavelengths for the STED beam is maintained. Although, the time-gated detection is mostly implemented using relatively expensive time-correlated-single-photon-counting (TCSPC) cards^10^,^24^,^32^,^33^,^34^,^36^,^37^, high speed gated photon counters^32^,^35^,^36^,^38^, fast-gated detectors^39^ and FPGA-based gated photon counters are fast emerging, thus offering soon cheaper alternatives to the TCSPC cards. Furthermore, the time gated-detection significantly reduces the peak STED beam intensity needed to reach effective sub-diffraction resolution^32^,^36^.

Since the same property at the base of the time-gated approach is also valid when the fluorophore is excited through two-photon absorption, it can be used to develop a novel 2PE-STED implementation (from now 2PE-gCW-STED microscope) with moderate cost and complexity. Although this implementation is straightforward, the smaller 2PE cross-section (with respect to the one-photon excitation counterpart), which may lead to a weak fluorescent signal, exacerbating with the major disadvantage of gCW-STED microscopy. That is time-gated detection reduces the fluorescence signal, hence, in a situation of weak signal and/or high background the images degrade in terms of signal-to-noise/background ratio (SNR and SBR) and the effective resolution improvement may vanish^24^. We have recently shown that the SNR of a gCW-STED image can be recovered through a multi-image deconvolution algorithm that takes advantage of the early-photons (before *T*_g_) normally discarded during the gCW-STED image formation process^40^. Whereas, the background, at least the one induced directly by the STED beam, can be subtracted by lock-in (synchronous detection) or filter based methods^36^,^41^,^42^. However, because they are based on a signal subtraction, the lock-in methods inevitably reduce the SNR. Hence, in the case of a weak fluorescence signal, typical of 2PE, they may drastically reduce the effective resolution of the proposed 2PE-gCW-STED microscope. We show that this problem is bypassed by including the possibility of direct background removal into the multi-image deconvolution algorithm. We implemented a 2PE-gCW-STED microscope operating in the visible range, thus compatible with the green and yellow fluorescent proteins, which currently represent the most popular markers for live applications. However, the same performance can be transferred to other wavelength regimes without any increases in the architecture complexity.

## Results

**Figure 1:**
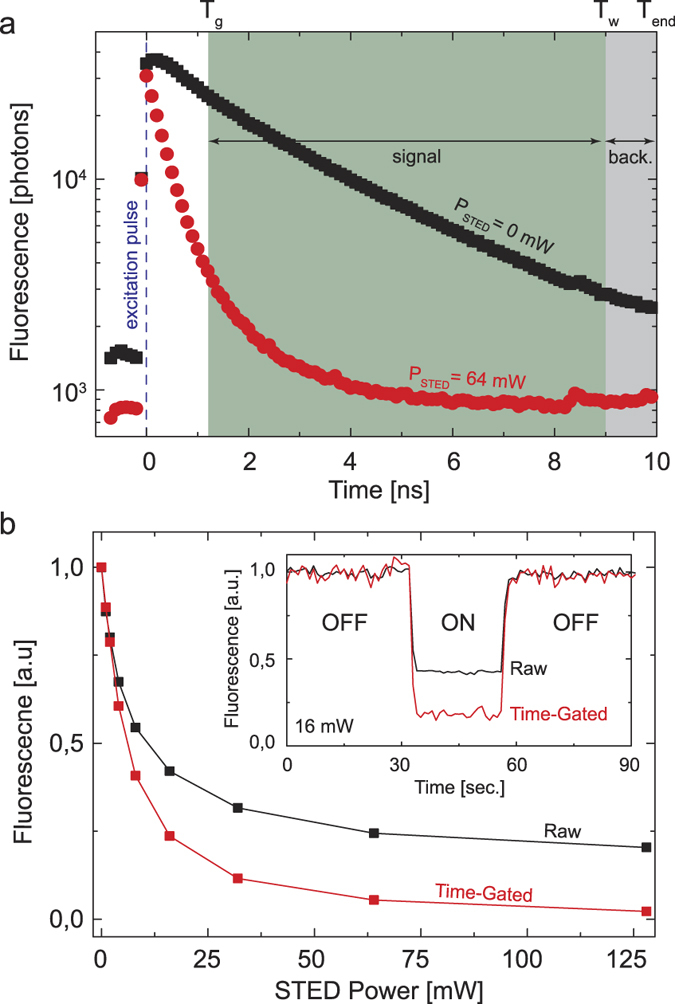
Principle of 2PE gCW-STED microscopy. (**a**) Histogram of the photon-arrival times (or TCSPC histogram) in a 2PE-STED experiment. The graph shows also the temporal characteristics of the gates used to implement the time-gated detection (green) and to estimate the anti-Stokes emission background (gray). (**b**) Raw (black) and gated (red, *T*_g _= 1.2 ns) depletion curves of Alexa Fluor 488 goat anti-mouse IgG diluted in PBS, λ_STED_ = 577 nm, λ_2PE_ = 760nm, P_exc_ = 15 mW. The inset shows a 90 seconds fluorescence recording in which the STED beam has been activated (P_STED_ = 16 mW) only in the middle interval. The On-Off contrast clearly enhances when the time-gated has been applied. The anti-Stokes emission background has been subtracted both from the raw and gated depletion curves/time traces.

The ability of a STED microscope to obtain sub-diffraction resolution is directly linked to the efficiency of silencing (switch-off) the fluorophores exposed to the stimulating photons. For a given intensity of the STED beam, the higher the probability of silencing a fluorophore, the higher the resolution attainable by the microscope. Since CW lasers generally provide lower (peak) intensity than pulsed lasers, the resolution of a system based on a STED beam running in CW is poorer than the pulsed counterpart. However, it has recently been shown than the switch-off efficiency, thus the effective resolution of systems based on STED beams running in CW can be improved without increasing the intensity, but instead by introducing a time-gated detection. This property can be readily explained by studying the time-dependent fluorescence emission probability of an excited-fluorophore^24^,^43^. Figure 1a shows the histogram of the photon-arrival times in a time-correlated-single-photon-counting (TCSPC) experiment (or TCSPC histogram) that represents an indirect measurement of the time-dependent fluorescence emission probability. The fraction to which the STED beam suppresses the fluorescence flux at time zero, i.e. immediately after the excitation events, is null but increases with continued action of the STED beam. Thus, collecting the fluorescence only after a certain time *T*_g_ from the excitation events improves the efficiency of silencing a fluorophore. It is also important to observe that the signal collected after a long delay (*T*_w_ > τ_fl_, with τ_fl_ the unperturbed excited-state lifetime of the fluorophore) is dominated by the fluorescence induced by the STED beam, namely the anti-Stokes emission background. This background appears as uncorrelated in the histogram of the photon-arrival times, because the STED beam runs in CW. This observation is the basis for different approaches developed to remove/subtract the anti-Stokes emission background in STED implementations based on the STED beam running in CW^42^,^44^. Certainly, tuning the STED beam of wavelength a long way from the absorption spectra of the fluorophore, avoids the anti-Stokes excitation background.

The improvement in silencing fluorophores by using time-gated detection is evident when measuring the so called depletion curve, namely the fraction of the suppressed fluorescence signal as a function of the STED beam power *P*_STED_ (Fig. 1b, Suppl. Fig. 1). Notably, the depletion curve measures the relative (with respect to the absence of stimulation photons) reduction of the recorded fluorescent signal and not the reduction of fluorescence emitted by the fluorophores. These two factors are equivalent in the case of full photon recording (raw), but different in the case of time-gated detection. We filtered both the raw and the time-gated depletion curves from the anti-Stokes emission background by using the TCSPC-based approach described by Coto Hernández *et al*^42^. In a nutshell, (i) we used the late time-bins (T_w_ < t < T_end_, grey area in Fig. 1a) of the TCSPC histogram to estimate the rate of the anti-Stokes emission background; (ii) we multiplied by this rate for the duration of the signal’s time-gate (green area in Fig. 1a) and we obtained the background to subtract, (iii) we subtracted the background from the gated fluorescent signal. For *P*_STED_ = 125 mW there is a residual fluorescent signal of 25%, but when the time-gated detection is applied (*T*_g_ = 1.2 ns) the fluorescent signal is completely suppressed. Furthermore, using the time-gated detection the fluorescent signal is suppressed down to 25% with only 12 mW instead of 125 mW. These results confirm that the benefits of time-gated detection are also valid under the 2PE regime. However, the lower cross-section of the two-photon absorption process (if compared with the one-photon cross-section) and the reduction of the overall fluorescent signal associated with the time-gated detection make the use of additional techniques to remove the anti-Stokes emission background crucial for many applications. In particular for such experiments in which tuning of the wavelength a long way from the fluorophore’s absorption is not straightforward. In this context, it is important to observe that the signal-to-background ratio (SBR) can be only partially optimized by increasing the power of the excitation beam, since photobleaching^45^ and fluorescence saturation (Suppl. Fig. 2) effects impose constraints.

**Figure 2:**
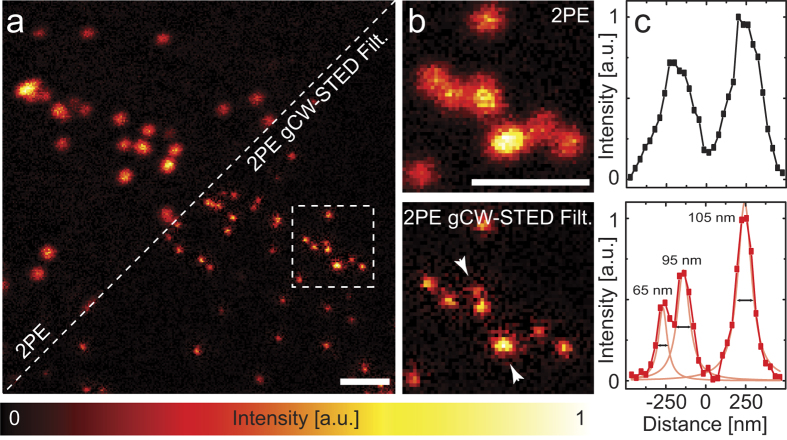
2PE gCW-STED imaging of fluorescent beads. (**a**) Comparison between 2PE (left-up corner) and filtered 2PE-gCW-STED (right-down corner) imaging. For the filtered 2PE-gCW-STED image the anti-Stoke emission background has been estimated directly from the TCSPC histogram and successively subtracted from the raw gated image. No deconvolution has been applied. (**b**) Magnification of the boxed areas. (**c**) Normalized intensity profiles along the arrows for 2PE (black) and 2PE-gCW-STED (red) images. *λ*_STED_ 577 nm, *P*_STED_ = 40 mW, *T*_g_ = 1.5 ns, *λ*_exc_ = 760 nm and *P*_exc_ = 15 mW. Scale bars 1 μm.

First, we demonstrated the benefits of time-gated detection in the context of 2PE-CW-STED microscopy by imaging sub-diffraction sized (40 nm) fluorescent beads (Fig. 2). Since the time-gated detection is implemented through a TCSPC, we estimated the anti-Stokes emission background image directly from the TCSPC histogram (as described for the depletion curve measurement^42^) without the need for a synchronous (or lock-in) detection scheme. We successively subtracted the estimated background from the raw 2PE-gCW-STED image to obtain the final image (Fig. 2a, right bottom corner).

Given an estimation of the background, subtracting it from the raw 2PE-gCW-STED is the most straightforward approach and it does not require the setting of any parameters. However, both the background estimation and the 2PE-gCW-STED image contain noise, thus a simple subtraction amplifies the noise in the final image. This noise amplification is not a problem when dealing with bright samples, i.e. 2PE-gCW-STED images with high SNR, but in the case of low SNR images the subtraction of the background can cancel-out the benefit of the time-gated detection. We addressed this problem by designing and implementing a dedicated image deconvolution algorithm based on a statistical formulation of the image formation process^46^. We have recently shown that multi-image deconvolution improves the SNR of a gated STED microscope. Indeed, the multi-image deconvolution combines the image formed by the usually discarded early photons (before *T*_g_) with the conventional gated STED image (photons after *T*_g_)^40^. Here, we extended the algorithm by including the (anti-Stokes emission) background as *a-priori* information in the linked image formation process. Hence, the multi-image algorithm automatically removes the (anti-Stokes emission) background and reassigns the early-photons to their original positions. The estimation of the anti-Stoke emission background image required in the multi-image deconvolution algorithm was obtained directly from the TCSPC histogram (as described for the depletion curve measurement^42^). Importantly, the algorithm is designed to impose a non-negative constraint on the restored image, thus avoiding the zero-clipping procedure that is usually applied in the subtractive methods.

**Figure 3:**
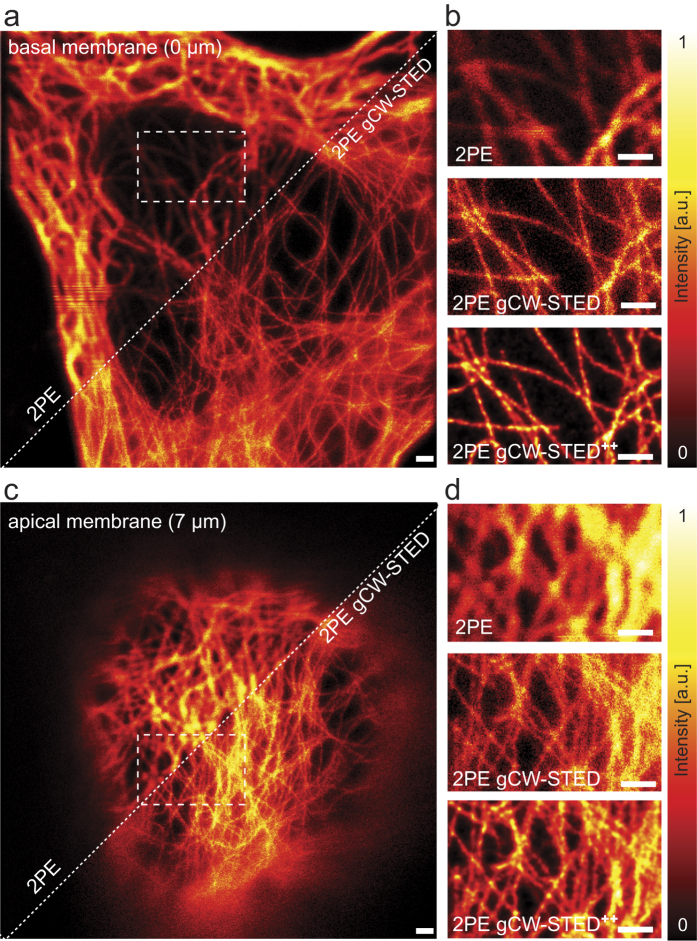
Comparison between 2PE and 2PE gCW-STED imaging of microtubules in a fixed HeLa cell. (**a,c**) Basal (**a**) and apical (**c**, 7μm deep) membrane imaged with 2PE (left-top corner) and 2PE-gCW-STED (right-bottom corner) microscopy. (**b,d**) Magnified views of the boxed area in **a** and **c**. The panels show a side-by-side comparison between 2PE (top), 2PE-gCW-STED (middle, no background subtraction and no deconvolution) and 2PE-gCW-STED^++^ (bottom, multi-image deconvolution). *λ*_STED_ 577 nm, *P*_STED_ = 40 mW, *T*_g_ = 1.5 ns, *λ*_exc_ = 760 nm and *P*_exc_ = 15 mW. Scale bars: 1 μm.

We demonstrated the synergy between the proposed gated 2PE-CW-STED implementation and the deconvolution algorithm by imaging the cytoskeleton at the basal (Fig. 3a, Suppl. Fig. 3) and the apical (7 μm depth, Fig. 3b, Suppl. Fig. 3) membrane of a Hela cell. Although, we observed stronger photo bleaching compared to one-photon excitation tested in similar conditions, the photo bleaching is highly localized on the focal plane, as expected from the non-linear nature of 2PE. Thanks to this condition, we could image the same cell at different depths. The comparison between the raw 2PE and the raw 2PE-gCW-STED images shows a marginal resolution improvement. Indeed, the reduction of the wanted fluorescence signal and the relative increase of the anti-Stokes emission background partially hide the expected resolution improvement. But, the application of the multi-images deconvolution (2PE-gCW-STED^++^) algorithm clearly enhances the SNR and at the same time removes the background.

The algorithm that we derived can be considered as a generalization of the well-known Richardson-Lucy (RL) algorithm^47^,^48^ (see also Supplementary Information). Similar to the RL algorithm our algorithm converges to a sparse (in the space domain) solution, that is after a large number of iterations the reconstruction consists of a set of bright spots over a black background, the so-called night-sky solution^46^,^49^. Although, this solution could be optimal for sparse samples, such as protein clusters; it is not optimal in general. Different regularization procedures have been proposed^46^,^49^,^50^, but all of them impose a different constraint on the reconstruction, thus again they cannot provide a solution for all kinds of sample. Besides using the early-photons to “regularize” the solution^40^, we avoid the night-sky effect by performing only a few iterations. In particular we applied only ∼ 10 iterations, which are enough to remove the (anti-Stokes emission) background and reassign the early-photos to their original positions.

It is also important to observe that, in stark-contrast with conventional microscopy, STED microscopy is a band-unlimited system, i.e., at least theoretically, all the sample frequencies are transmitted by the system and in practice only the noise distorts the high-frequencies. In a nutshell, the noise and not the diffraction, represents the ultimate limiting factor for the spatial resolution^51^. For these reasons, image deconvolution can play a fundamental role in recovering frequencies well below the diffraction limit, which are transmitted by the system but hidden by the noise^51^. On the contrary, when deconvolution is applied to conventional microscopy, the sub-diffraction frequencies recovered by the algorithm are strictly linked to the *a-priori* information introduced by the algorithm and they could be associated to artifacts.

## Discussion

We have presented a novel 2PE-STED implementation based on time-gated detection and STED beam running in CW. The use of the STED beam running in CW tempers the complexity and the cost of the architecture and furthermore gives a large flexibility on the choice of the wavelength-regime at which the system can operate. Besides the technical benefits, the time-gated detection substantially enhances the ability to silence a fluorophore, which is critical for obtaining effective sub-diffraction resolution at moderate STED beam intensity.

However, the benefits of the time-gated detection come along with a reduction of the fluorescence signal that forms the image. Indeed, even useful fluorescence photons stemming from the doughnut centre are discarded by the time gated detection. As a matter of fact, for experiments with a limited budget of fluorescence photons or non-negligible background, the SNR and SBR reduction can cancel-out the expected resolution improvement. In this work we have mitigated this limitation by introducing a tailored deconvolution algorithm that simultaneously improves the SNR and the SBR by reassigning the discarded fluorescent photons to their origin and by removing the anti-Stokes emission background. The algorithm requires an estimation of the background. We demonstrated that a good estimate is obtained from the TCSPC histogram; however the algorithm can also work in combination with other background estimation methods, such as the synchronous (or lock-in) detection^36^,^41^. Thus, if an estimation of the background is provided, the proposed algorithm is compatible with pulsed STED implementations (one- or two-photon excitation). In the context of pulsed 2PE-STED (2PE-pSTED) implementations, it is important to highlight that novel pSTED microscopes based on externally triggered picoseconds/nanoseconds (500–1500 ps) lasers to provide the STED beams, have recently been demonstrated^3^,^52^. Nevertheless, these implementations rely on one-photon-excitation architectures, they can be straightforwardly extended to the 2PE regime: the STED beam provided by the synchronized Ti:Sapphire laser (plus an OPO in case of visible range implementation) could be substituted for these new compact and cheaper laser systems. Furthermore, the synchronization with the Ti:Sapphire laser providing the excitation beam becomes trivial. Notably, these picoseconds/nanoseconds lasers do not supply wavelength tunability, so far, but they cover both the visible and the near-infrared region. Whilst the first region is necessary for applications requiring visible fluorescent proteins (such as GFP and YFP), the later are better suited for deep-imaging applications. The possibility of using long pulses (nanoseconds) could also help to reduce the photodamage. Indeed, long pulses reduce the peak intensity and most of the photodamage effects scale supra-linearly with the peak intensity^53^. However, if the length of the pulses (T_STED_) is no longer negligible with respect to the lifetime of the fluorophores (T_STED_ ∼ τ), the time-gated detection also becomes important in the all-pulsed implementation^24^. Time-gating removes the fluorescent signal that occurs during the action of the STED beam pulses. As a matter of fact, the algorithm presented in this work is relevant for any STED implementations.

We show that image deconvolution successfully improves the SNR and the SBR of the 2PE-gCW-STED microscopy images. Furthermore, we expect similar results also for images obtained from other STED microscopy implementations. However, such image deconvolution methods are hardly (if ever) applicable for spectroscopy STED measurement, such as STED fluorescence correlation microscopy (STED-FCS)^12^,^54^, essentially because the spatial context (neighborhood) is normally ignored. An alternative to image deconvolution in the framework of CW-STED-FCS could be the recent approach proposed by Lanzanò *et al.*^44^. Here, by using the nanosecond temporal dynamics of the fluorescence signal, all the fluorescent photons stemming from the center of the detection volume can be isolated from the peripheral and the background photons.

The demand for deep imaging with sub-diffraction resolution is growing exponentially in many fields, and in particular in neuroscience. The potential of 2PE-STED microscopy to investigate the neuronal morphology has already been demonstrated; however the number of studies using this approach is very limited. We believe that this limitation derives from the complexity and cost of the current 2PE-STED implementations. For these reasons, the 2PE-gCW-STED implementation, shown in this work, and the gated 2PE-pSTED implementation discussed above, could effectively open a wide dissemination of STED microscopy in neuroscience.

## Methods

### Gated 2PE-CW-STED Microscope

We implemented the 2PE-gCW-STED microscope on top of an existing one-photon excitation gCW-STED microscope. Briefly (see Suppl. Fig. 4), a femtosecond mode-locked Ti:Sapphire laser (Chameleon, Vision II, Coherent) provided the 80 MHz repetition rate beam to induce 2PE. We tuned the wavelength of the laser to λ_exc_ = 760 nm in order to optimize the two-photon cross-section of the Alexa 488 dye in water. The pre-chirping unit integrated in the laser box allowed the optimization of the 2PE efficiency by compensating for the pulse-broadening introduced in the 2PE beam path from the lenses, the mirrors and the acoustic optic modulator (AOM, AA Opto-Electronic MCQ80-A1,5-IR). An optical pumped semiconductor laser (OPSL) at λ_STED_ = 577 nm (Genesis CX STM-2000, Coherent) provided the STED beam running in CW^55^. We used an AOM to control the power of the 2PE beam, and a rotating half-wave-plate (RAC 4.2.10, Bernhard Halle Nachfl.) combined with a fixed Glan-Thompson polarizing prism (PGT 1.10, Bernhard Halle Nachfl.) to control the power of the STED beam. We collinearly aligned the STED and the 2PE beam using two dichroic mirrors (2P-Beamsplitter 720 dcxxr and z-560-sprdc, AHF analysentechnick). The collinearly aligned beams were deflected by two galvanometric scanning mirrors (6215HM40B, CTI-Cambridge) and directed toward the objective lens (HCX PLAPO 100**×**/1.40 − 0.70 Oil, Leica Microsystems) by the same set of scan and tube lenses as the ones used in a commercial scanning microscope (Leica TCS SP5, Leica Microsystems). We obtained a doughnut-like diffraction pattern at the focus, by passing the STED beam though a polymeric mask imprinting 0-2π helical phase-ramps (VPP-A1, RPC Photonics) and by imposing circular polarization to the beam in the back-aperture of the objective lens. We obtained the circular polarization by carefully rotating an achromatic half-wave retardation plate (RAC 4.2.10,Bernhard Halle Nachfl.) and an achromatic quarter-wave retardation plate (RAC 3.4.15, Bernhard Halle Nachfl.), located between the phase mask and the dichroic mirror. The fluorescence light was collected by the same objective lens, de-scanned, and passed through the dichroic mirrors as well as through a fluorescence band pass filter (ET Bandpass 525/50 nm, AHF analysentechnik) before being focused (focal length 30 mm, AC254-030-A-ML, Thorlabs) into a fiber pigtailed single photon avalanche diode (SPAD) (PDF Series, MicroPhotonDevice). The graded index multimode fiber with 62.5 μm core of the SPAD acted as a confocal pinhole of 2.3 times the size of the back-projected Airy disk at 520 nm. It is worth nothing that in 2PE microscopy, the pinhole is not necessary, thus a non-de-scanned implementation is usually preferred. Indeed, the out-of-focus fluorescence is inherently “rejected” by the non-linearity of the excitation process. However, in 2PE-STED microcopy, if the STED beam generates a substantial amount of out-of-focus anti-Stokes emission background, the pinhole helps significantly. Conversely, a small pinhole could drastically reduce the signal.

A time-correlated-single-photon-counting-card (TCSPC) (SPC-830, Becker & Hickl), synchronized with the reference signal provided as output by the Ti:Sapphire laser, counted the photons and measured their arrival-time. All the power values for the excitation and STED beams were measured at the back aperture of the objective lens.

Every imaging operation was automated and managed by the software Imspector (Imspector, Max Planck Innovation).

### Multi-Image Deconvolution Algorithm

We implemented a multi-image deconvolution algorithm that integrates the possibility of removing a background component from the restored image. The multi-image structure of the algorithm allows the fusion of the late-photons (after *T*_g_) image with the early-photons (before *T*_g_) image. The latter is normally rejected because it does not contain high-resolution information (i.e. high spatial frequencies). However, it has been shown that the early-photons image can be used to improve the SNR of gated STED microscopy^40^. Starting from the multi-image deconvolution algorithm derived by Castello *et al.*^40^, we included the possibility of introducing prior information about the background of each of the images into the deconvolution problem. The new algorithm reads (see Supplementary Information):


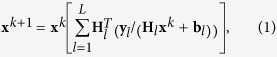


where (i) **x**^k^ denotes the restored images at the k-th iteration; (ii) **y**_l_ denotes the l-th image associated to the l-th PSF, *h*_l_; (iii) **H**_l_ is the matrix notation for the convolution operator associated to the l-th PSF, *h*_l_; (iv) **b**_l_ is the background image associate of the l-th image. In the contest of this paper, only two images (L = 2) need to be fused, the late- (l = 1) and the early-photons (l = 2) images, respectively. It is important to note that following the statistical derivation of the multi-image Richardson-Lucy algorithm each pixel of the background image *b*_l_ represents the mean (expected) value of a random (Poissonian) variable^24^,^40^. Whereas it is difficult to assess the mean value of the anti-Stokes emission background it is possible to have a realization (measure) of it. In particular, this realization can be obtained from the “close” phase of a synchronization (lock-in) measurement or, in the case of gCW-STED implementation, from the histogram of the photon-arrival times provided by the TCSPC measurement. Indeed, in this later implementation the anti-Stokes emission photons are uncorrelated with respect to the pulses of the excitation beam. Here we choose the second approach: In particular, for each pixel of the STED image we used the last bins [*T*_w_.*T*_end_] of the histogram of the photon-arrival times to have a realization of the background **b**_r_^42^. Successively, we estimated the expected value of the background associated to the l-th image as follows


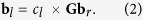


where **G** is a Gaussian operator whose sum is normalized to 1 to preserve the total counts of the background image *b*_r_ and *c*_l_ is a weight factor linked to the time-length of the gated associated to the the l-th image, i.e. *c*_1 _= *T*_g_/(*T*_end_ − *T*_w_) and *c*_2_ = (*T*_w_ − *T*_g_)/ /(*T*_end_ − *T*_w_).

It is very important to note that, due to the multiplicative structure of the algorithm (see Eq. 1), if the algorithm is initialized with a non-negative image, the reconstructed image is also non-negative. In this work **x**^0^ is a constant image with total flux equal to the sum of the early- and late-photons image minus the background images **b**_*l*_.

We estimated the point-spread-functions of the late- and early-photon images directly from the 2PE and 2PE-CW-STED images following the protocol described in Castello *et al.*^40^.

**Samples.** We diluted the fluorescent beads (**~**40 nm diameter, Yellow-Green, Invitrogen) in water by 1:3000 (v/v), we dropped the dilute solution onto a poly-L-lysine (Sigma) coated glass coverslip, we waited 10 min, we washed it with water and we dried the coverslip by blowing nitrogen onto it. Finally, we mounted the coverslip with a special medium (Mounting Medium, Invitrogen) and we observed it with the microscope.

For immunofluorescence assays, we cultured HeLa cells (derived from a human cervix carcinoma) on glass coverslips (18 mm diameter) in Dulbecco’s modified Eagles medium (DMEM) (Invitrogen) supplemented with 10% fetal bovine serum (FBS) (Invitrogen), 100 IU/ml penicillin and 100 μg/ml streptomycin (Invitrogen) at 37 °C in a humidified atmosphere containing 5% CO2 for 24 h. We rinsed the plated cells with phosphate-buffered saline (PBS) (0.1 M, pH 7.4) and fixed them by incubation in 4% formaldehyde in PBS (0.1M, pH 7.4) for 15 min. We washed the fixed cells with PBS (0.1 M, pH 7.4) and permeabilized for 30 min at room temperature with 3% normal bovine serum albumin (BSA) and 0.1% Triton-X-100 in PBS (0.1 M, pH 7.4). We incubated the cells with the monoclonal mouse anti-**α**-tubulin antiserum (Sigma Aldrich) diluted in PBS plus 0.1% Triton-X-100 and 3% BSA for 1 h at room temperature. The **α**-tubulin antibody was revealed using Alexa Fluor 488 goat anti-mouse IgG (1:500, Molecular Probes). The coverslips were rinsed in PBS (0.1 M, pH 7.4) and then placed in an open-bath imaging chamber containing PBS (0.1 M, pH 7.4) and observed with the microscope.

## Additional Information

**How to cite this article**: Coto Hernández, I. *et al.* Two-Photon Excitation STED Microscopy with Time-Gated Detection. *Sci. Rep.* **6**, 19419; doi: 10.1038/srep19419 (2016).

## Supplementary Material

Supplementary Information
