# Supplementary material for: Two-Photon Excitation STED Microscopy with Time-Gated Detection
Source: Sci Rep. 2016 Jan 13;6:19419. doi: 10.1038/srep19419 (PMC4725939; doi:10.1038/srep19419)
Supplement: Supplementary Information [file srep19419-s1.pdf]

Supplementary Information

Two-Photon Excitation STED Microscopy with Time-Gated Detection

Iván Coto Hernández[1,2,5,+], Marco Castello[1,3,+], Luca Lanzanò[1], Marta d'Amora[1], Paolo Bianchini[1], Alberto Diaspro[1,2,4,] and Giuseppe Vicidomini[1,*]*

[1]Nanoscopy, Nanophysics, Istituto Italiano di Tecnologia, Via Morego 30, 16163 Genoa, Italy,

[2]Department of Physics, University of Genoa, Via Dodecaneso 33, 16146, Genoa, Italy,

[3]Department of Informatics, Bioengineering, Robotics and Systems Engineering, University of Genoa, Via Opera Pia 13, 16145, Genoa, Italy, [4] Nikon Imaging Center, Istituto Italiano di Tecnologia, Via Morego 30, 16163 Genoa, Italy. [5] Currently with the Institut des Sciences Moléculaires d'Orsay, CNRS, Université Paris Sud, Université Paris-Saclay, F91405, Orsay Cedex, France

[+]these authors contributed equally to this work

*alberto.diaspro@iit.it or giuseppe.vicidomini@iit.it

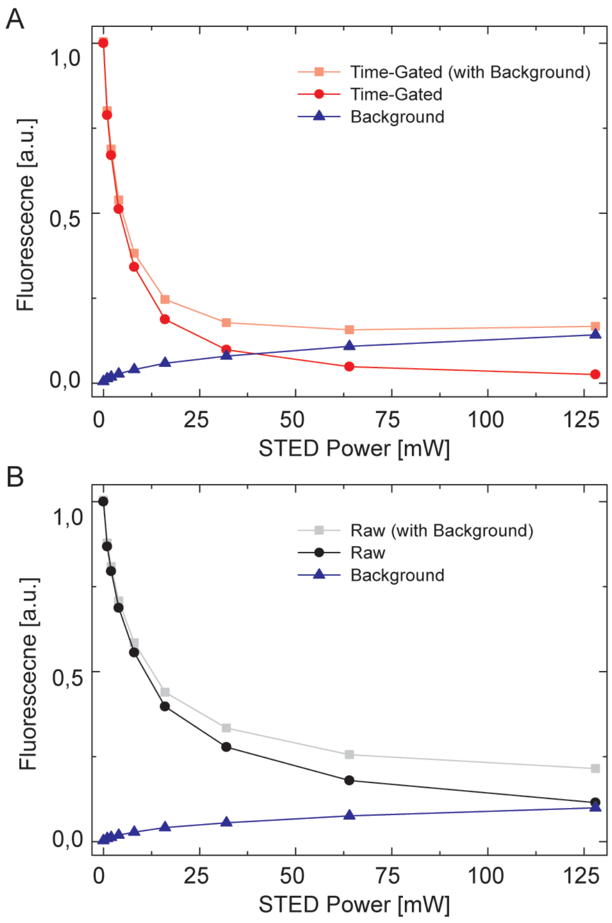

Suppl. Fig. 1. Depletion curves with and without background subtraction. (a) Time-gated depletion curves of Alexa Fluor 488 goat anti-mouse IgG diluted in PBS after (red) and before (light red) the anti-Stokes emission background subtraction. (b) Raw depletion curves of Alexa Fluor 488 goat anti-mouse IgG diluted in PBS after (black) and before (gray) the anti-Stokes emission background subtraction. The anti-Stokes emission background (blue) was estimated from the TCSPC histogram of each measurement and successively subtracted [1].

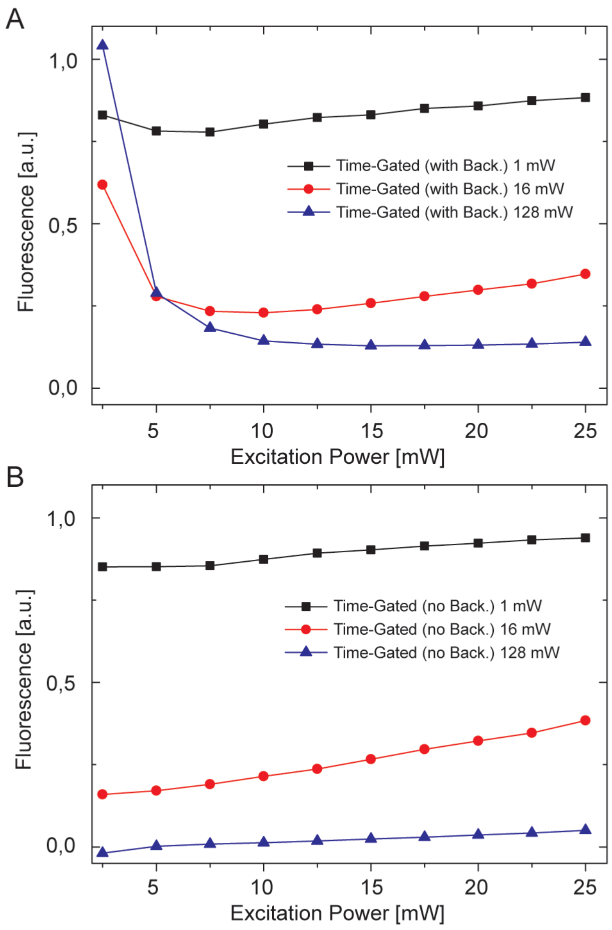

Suppl. Fig. 2. Switching-off contrast as a function of the excitation power. (a) Residual fluorescence after the time-gated detection as a function of the power of the excitation beam for different power of STED beam. At low excitation power (< 15 mW) the anti-Stokes emission background dominates over the fluorescence induced by the excitation beam, thus reducing the efficiency to silence fluorophores. Similarly, the efficiency decreases for increasing excitation power (> 15 mW) because the saturation of the excitation transition. Thus, it is important to tune the excitation power in order to maximize the fluorescence signal with respect to the background and to avoid saturation phenomena. (b) Same as (a) but with the subtraction of the background. As expected the subtraction of the anti-Stokes emission background does not remove the lost in efficiency caused by the saturation of the excitation transition.

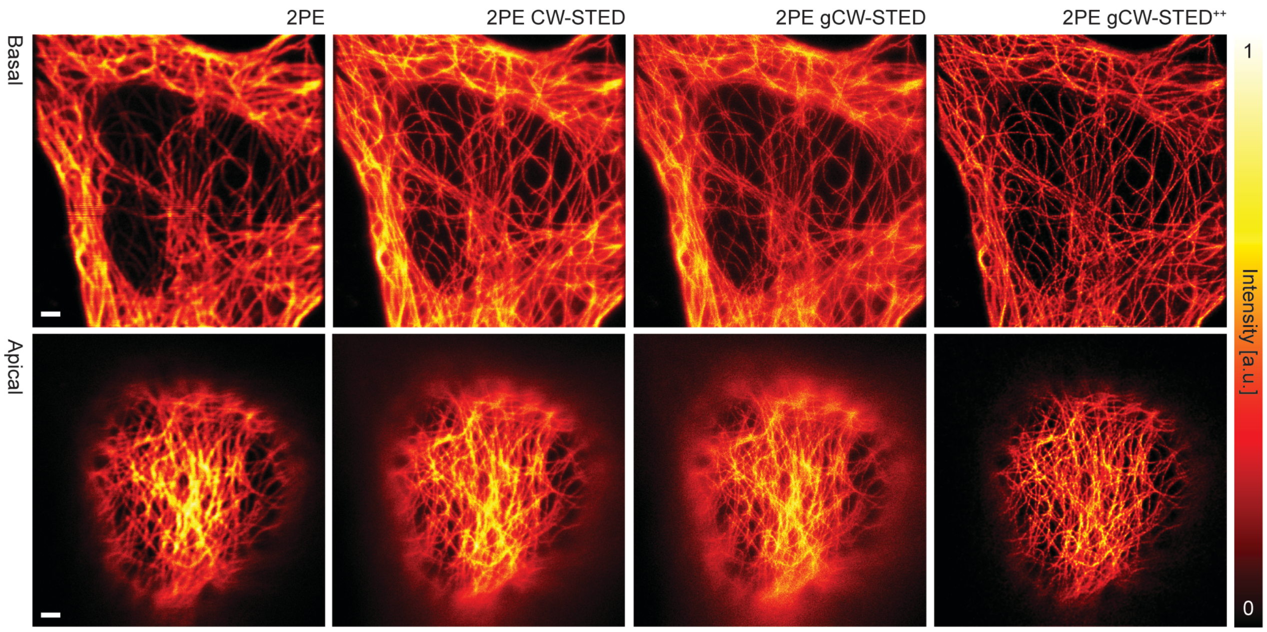

Suppl. Fig. 3. Comparison between 2PE and 2PE gCW-STED imaging of microtubules in a fixed HeLa cell. The images shown in Fig. 3 are selections of this panel. Scale bars: 1 µm.

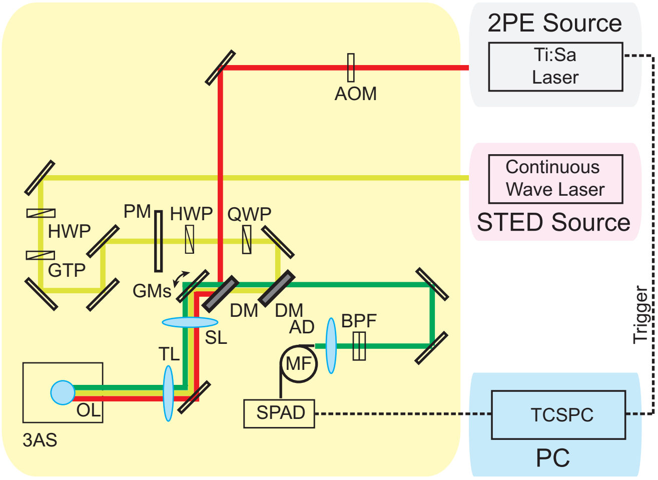

Suppl. Fig. 4. Experimental setup for 2PE gCW-STED microscopy. AOM: Acousto-optic modulator; HWP: half-wave plate; GTP: Glan–Thompson polarizer; PM: phase mask; QWP: quarter-wave plate; DM: dichroic mirror; GMs: galvometer mirrors; SL: scanning lens; TL: tube lens; OL: objective lens; 3AS: three-axis stage; BPF: band-pass filter; AD: achromatic doublet; MF: multi-mode fiber; SPAD: single-photon avalanche diode; TCSPC: time-correlated-single-photon-counting card; PC: personal computer

Multi-Image Deconvolution Algorithm

The full derivation of the multi-image algorithm, including the introduction of a generic background, has been already described in Castello et al. [2]. For sake in simplicity, here, we report the main equations.

We first formulate the image process formation from a statistical point-of-view. We assume that for any pixel $i = 1,...,n$ of any image $\mathbf{y}_l$ with $l=1,...,L$, the value $(\mathbf{y}_l)_i$ is the realization of an independent Poisson random variable $Y_{i,l}$ with mean value $(\mathbf{H}_l\,\mathbf{x} + \mathbf{b}_l)_i$, i.e.

$$Y_{i,l} \sim Poisson\{(\mathbf{H}_l\mathbf{x} + \mathbf{b}_l)_i\}, \qquad (1)$$

where: (i) $\mathbf{x}$ is a discrete mapping of the fluorophore's concentration in the sample; (ii) $\mathbf{H}_l$ is the discrete notation for the convolution operator associated to the l-th PSF h_l; (iii) $\mathbf{b}_l$ denotes the background map for the l-th images. Notably, the assumption of Eq. 1 is a consequence of the fact that both the photons registered and generated from the sample and the photons registered and considered as background are realization of independent Poisson random variables, thus the sum is a Poisson random variable as well. In the next, we well considered $\mathbf{b}_l$ as *a-priori* information. The protocol how to derive an estimation of the mean for the anti-Stokes emission background is described in the main text.

Thanks to the independence of each random variable $Y_{i,l}$, we can write the probability density distribution as

$$P_Y(\mathbf{y}|\mathbf{x}) = \prod_{l=1}^{L} Y_l = \prod_{l=1}^{L}\prod_{i=1}^{N} Y_{i.l} = \prod_{i,l} \frac{e^{-(\mathbf{H}_l\mathbf{x}+\mathbf{b}_l)_i}(\mathbf{H}_l\mathbf{x}+\mathbf{b}_l)_i}{(\mathbf{y}_l)_i!}, \qquad (2)$$

where $\mathbf{y} = \{\mathbf{y}_l\}$.

Since we assume to know the probability density distribution $P_Y(\mathbf{y}|\mathbf{x})$ of the data and since the unknown object $\mathbf{x}$ appear as a set of parameters, the problem of restoring $\mathbf{x}$ can be treated as a classical problem of parameters estimation, which is usually solved through the maximum likelihood approach. In our case, this approach consists in introducing the likelihood function

$$L_{\mathbf{y}}^{Y}(\mathbf{x}) = P_Y(\mathbf{y}|\mathbf{x}), \qquad (3)$$

then, the ML-estimate of the unknown object is any object $\mathbf{x}^*$ that maximize the likelihood function with respect to $\mathbf{x}$

$$\mathbf{x}^* = \mathrm{argmax}_{\mathbf{x}}\, L_{\mathbf{y}}^{Y}(\mathbf{x}). \qquad (4)$$

This maximization problem can be transformed into a minimization problem by considering the negative logarithm of the likelihood function

$$J(\mathbf{y};\mathbf{x}) = \sum_{i,l}\left((\mathbf{y}_l)_i ln\frac{(\mathbf{y}_l)_i}{(\mathbf{H}_l\mathbf{x}+\mathbf{b}_l)_i} + (\mathbf{H}_l\mathbf{x}+\mathbf{b}_l)_i - (\mathbf{y}_l)_i\right), \qquad (5)$$

that is the well-known Kullback-Leibler (KL) divergence of the vector $\mathbf{H}_l\,\mathbf{x} + \mathbf{b}_l$ from the vector $\mathbf{y}$.

Minimization of the KL divergence under a positive constraint $\mathbf{x} > 0$ leads to the following iterative algorithm

$$\mathbf{x}^{k+1} = \mathbf{x}^{k}\left[\sum_{l=1}^{L} \mathbf{H}_l^T\left(\mathbf{y}_l/(\mathbf{H}_l\mathbf{x}^k + \mathbf{b}_l)\right)\right], \tag{6}$$

which can be considered as an extension of the Richarson-Lucy algorithm for solving the multi-image deconvolution problem. Indeed, for $L = 1$, the algorithm reduces to the well-known Richardson-Lucy algorithm.

1. Coto Hernàndez I, Peres C, Cella Zanacchi F, d'Amora M, Christodoulou S, Bianchini P*, et al.* A new filtering technique for removing anti-Stokes emission background in gated CW-STED microscopy. *J Biophotonics* 2014; **7**(6)**:** 376-380.
2. Castello M, Diaspro A, Vicidomini G. Multi-images deconvolution improves signal-to-noise ratio on gated stimulated emission depletion microscopy. *Appl Phys Lett* 2014; **105**(23)**:** 234106.
